# Killing and letting die: consequence responsibility in guidance control

**DOI:** 10.1007/s11017-026-09744-7

**Published:** 2026-02-18

**Authors:** Mingrui Zhao

**Affiliations:** Faculty of Arts, Monash University, Clayton, VIC 3800 Australia

**Keywords:** Killing and letting die, Moral responsibility, Guidance control, Consequence responsibility

## Abstract

This paper reassesses the alleged moral difference between killing and letting die by shifting the analysis from permissibility to the allocation of consequence responsibility. Within Fischer and Ravizza’s guidance-control framework, responsibility is two-staged: agents are conduct-responsible when their behaviour issues from a mechanism they own that is at least moderately reasons-responsive; and they are consequence-responsible when, in addition, the death arises along a non-deviant, normatively intelligible route from that behaviour. I then operationalise the second stage via a disciplined counterfactual-subtraction procedure that is neutral between actions and omissions and that distinguishes consequence-particulars from consequence-universals. Applied to canonical contrasts, the framework yields patterned results: where both killing and letting die satisfy (or both fail) the same responsibility conditions, they are moral equals with respect to the death; where they come apart, any asymmetry tracks ownership, reasons-responsiveness, or decisive causal contribution rather than the surface doing/allowing label. The upshot is a unified, practice-usable standard for foreseeable-death contexts in end-of-life medicine and clinical governance, clarifying documentation, role delineation, and the evidential focus for retrospective appraisal without presupposing a default moral privilege for omissions.

## Introduction

Legal doctrine has long treated letting die as less morally and legally problematic than direct killing. In English common law, the House of Lords in *Airedale NHS Trust v Bland* characterised withdrawal of life-sustaining treatment as an omission rather than a killing, insulating clinicians from homicide liability [1]. Civil-law and clinical-ethics discussions report a parallel asymmetry: in most jurisdictions, withholding or withdrawing treatment is accepted under specified conditions, while active life-ending remains prohibited [2]. Rachels’s celebrated Smith-and-Jones case challenges this orthodoxy: when outcome and motive are held constant, the causal mode—doing versus allowing—seems to make no moral difference [3]. Subsequent theory largely agrees that what matters is not bodily motion but the structure of agency and causal contribution. Quinn defends a morally significant doing/allowing distinction by analysing positive versus negative agency in rescue-type cases [4]. McMahan develops finely grained criteria in cases of withdrawing aid, arguing that many such cases are best classified as letting die rather than killing [5]. More recent work stresses limits and heuristics rather than sharp principles: Räsänen and Häyry argue that Rachels’ contrast strategy does not establish the permissibility of active euthanasia and highlights methodological constraints on what philosophical bioethics can show [6]; Colombo models the doing/allowing contrast as a fallible moral heuristic—useful yet prone to misfire in atypical frames [7]; and Riisfeldt places the killing-versus-letting-die divide as the first and highly contested axis in a value-neutral taxonomy of end-of-life practices [8]. If there is indeed no intrinsic moral difference between killing and letting die, persisting in a legal-ethical privileging of omissions risks systematic disparity: agents bearing comparable responsibility for a foreseen death may face divergent censure solely because of the causal route. Clarifying whether the doing/allowing distinction tracks any morally relevant difference is therefore both philosophically urgent and practically consequential.

This article examines the killing/letting-die distinction through the lens of moral responsibility rather than permissibility. Although the debate is typically framed in terms of what is allowed, it also bears on whether agents who secure the same outcome by different causal modes bear the same kind of responsibility. Because both killing and letting die typically culminate in death, the central question is whether differences in mode of conduct—doing versus allowing—reshape how responsibility is attributed. A responsibility-focused analysis therefore complements, and may correct, purely permissibility-focused approaches. To structure this analysis, I adopt Fischer and Ravizza’s guidance-control framework, which grounds responsibility in the agent’s ownership of a moderately reasons-responsive mechanism and in her causal contribution to the outcome [9].

The argument unfolds in four steps. The first section explains why a responsibility-centred analysis is indispensable for end-of-life disputes in which death is known or reasonably foreseeable. I separate forward-looking guidance questions (what one ought to do under outcome-uncertainty) from backward-looking moral judgement (how responsibility for a known or foreseeable death should be allocated), and I argue that the latter cannot be reduced to permissibility alone. The second section develops the guidance-control framework and a two-stage account of responsibility. At the first stage, agents are conduct-responsible when their conduct issues from a mechanism they properly “own” and that is at least moderately reasons-responsive. At the second stage, agents are consequence-responsible when, in addition, their conduct contributes to the death along a stable, non-deviant, normatively intelligible route. The third section operationalises this second stage with a disciplined counterfactual-subtraction test that treats consequence-particulars and consequence-universals differently and respects nearest-world and non-deviation constraints. And the fourth section applies the framework to canonical pairs of killing and letting die. It shows that where both modes satisfy the same conditions for consequence responsibility, no further moral difference remains to be explained; where they come apart, any residual asymmetry tracks differences in ownership, reasons-responsiveness, or decisive causal contribution rather than the surface contrast between acting and omitting.

## Permissibility and responsibility in moral judgement

Debates over whether killing and letting die differ morally often involve cases with a shared structure: the conduct under evaluation—acting or omitting—occurs within a sequence whose fatal outcome is known or reasonably foreseeable. In such foreseeable-death contexts, permissibility cannot do all the work; a complete appraisal also requires allocating consequence responsibility for the death. By “moral guidance” I mean forward-looking deliberation under outcome-uncertainty; by “moral judgement” I mean retrospective appraisal where the outcome is known or suitably foreseeable. The fundamental distinction, then, turns on the epistemic status of outcomes at the time of decision. The present section clarifies why, in this retrospective sense, moral judgement must address the allocation of responsibility in addition to questions of permissibility.

Many everyday moral questions foreground guidance because the relevant outcomes cannot be reliably predicted in advance. The familiar puzzle of whether to tell a “white lie” illustrates this: honesty may risk hurting someone’s feelings, while deception may undermine trust; reasonable agents disagree about which option better promotes value—utility for utilitarians [10–12] or the demands of good will and the highest good for Kantians [13]. In such contexts of outcome-uncertainty, the central task is to assess the permissibility of competing actions so as to guide deliberation, rather than to allocate responsibility for a particular result.

Retrospective moral judgement is different. It evaluates a particular act-token in a context where the relevant facts are known or reasonably foreseeable, and where the task is to determine whether, and to what extent, the agent can be held responsible for the ensuing outcome. Consider, for example, a case drawn from real events during the 2023 Esk Valley flood in New Zealand. From an abstract moral-guidance perspective, disobeying a military order appears impermissible on both utilitarian and Kantian grounds: it threatens coordinated effectiveness and fails the test of universalisation. Yet the news report documents a situation in which several Army Reserve soldiers ignored standing instructions and redirected efforts toward rescuing civilians who were in immediate danger of drowning [14]. Viewed retrospectively—once the outcome is known—their decision is widely regarded as courageous, prudent, and morally admirable. This contrast illustrates why retrospective moral judgement cannot rely on permissibility alone. When the outcome is known or reasonably foreseeable, our evaluation shifts from asking whether the soldier ought to have followed the rule ex ante to asking whether the actual deaths or survivals are, in the relevant sense, attributable to the soldier’s conduct. The moral shift is therefore not simply from “wrong” to “right,” but from a rule-focused appraisal under uncertainty to a responsibility-focused judgement once the consequences have become clear.

A helpful way to mark this contrast is through the legal analogy between legislation and adjudication. Moral guidance is legislation-like: prospective, relatively general, and oriented toward establishing action-rules that can sensibly direct conduct under conditions of uncertainty. Moral judgement is adjudication-like: retrospective, particularised, and sensitive to the evidential contours of the actual case. It involves two distinct questions. First, a permissibility question: was the conduct, given its reasons and circumstances, allowed, required, or forbidden? Second, a responsibility question: given what actually happened—or was reasonably foreseeable—does the agent bear responsibility for the outcome? What matters here is not merely whether the action was permissible in the abstract, but whether the agent’s contribution counts as a morally attributable cause emerging from a mechanism she properly owns and that is at least moderately responsive to reasons. The analogy therefore clarifies that guidance and judgement address different evaluative tasks: one centres on rule-setting under uncertainty, the other on attributing responsibility once the outcome is fixed.

Assessment in foreseeable-death medicine therefore proceeds along two axes. The permissibility axis asks whether the conduct—considered in light of its reasons and circumstances—was allowed, required, or forbidden. The responsibility axis asks whether the agent’s contribution to the death qualifies as a morally attributable cause along a non-deviant, normatively intelligible route. Keeping these axes distinct in practice guards against intuitive drift, preventing familiar labels such as killing and letting die from solidifying into moral verdicts without a structured appraisal of reasons and contributions.

To render the responsibility axis tractable, the remainder of the paper adopts John Martin Fischer and Mark Ravizza’s guidance-control framework. On this view, agents are responsible for their actions when the behaviour issues from an owned, moderately reasons-responsive mechanism; they are responsible for consequences when, in addition, their behaviour contributes to the outcome along a stable, non-deviant pathway that makes the result “up to” the agent. When applied to killing and letting die, this framework distinguishes mere causal involvement from morally attributable contribution and clarifies why the doing/allowing contrast has limited normative force once responsibility is properly allocated.

Methodologically, separating the two axes yields three payoffs. First, it disciplines retrospective appraisal by specifying in advance what is being evaluated, reducing the influence of hindsight and outcome bias in foreseeable-death contexts. Second, it offers a neutral descriptive vocabulary—acting, omitting, enabling, authorising—for heterogeneous forms of involvement without presupposing default moral weight for either activity or passivity. Third, and central to this paper’s contribution, it reframes the killing/letting-die debate through a responsibility-centred lens: instead of asking whether the two categories differ as such, it asks which differences in responsibility underwrite their divergent moral profiles. This shift provides a unified and operational way of analysing cases that have long resisted clean categorisation. The rest of the paper keeps these axes distinct, treats labels like “killing” and “letting die” as provisional placeholders until responsibility is attributed under guidance control, and operationalises consequence-responsibility via a counterfactual-subtraction procedure before turning to canonical pairs of killing and letting die.

## Responsibility for consequences in guidance control

Fischer and Ravizza’s guidance-control framework holds that a death is attributable to an agent only when two broad conditions are met. The first concerns the agent’s conduct: the behaviour must arise from capacities and mechanisms that the agent has, over time, taken responsibility for—mechanisms she recognises as her own, rather than products of manipulation, bypassing, or external intrusion. Although Fischer and Ravizza label this condition “responsibility for action,” what matters for present purposes is the modality of the behaviour rather than the metaphysics of action; I therefore refer to it as conduct-responsibility. Conduct is attributable when it issues from a mechanism that is both the agent’s own, in the historical sense developed later in their account [9, pp. 299–231], and moderately reasons-responsive, meaning that it displays a recognisable pattern of receptivity to reasons together with at least minimal reactivity across nearby possibilities [9, pp. 79–82]. This threshold is intentionally modest: it excludes pathological or bypassed mechanisms but does not require ideal rationality.

The second condition concerns how the world responds to the conduct. A death is attributable only when the outcome unfolds along a route that is stable, non-deviant, and normatively intelligible—what Fischer and Ravizza call a responsive sequence, consisting of an ‘inner mechanism’ leading to the bodily movement and an ‘outer path’ leading from that movement to the external event [9, pp. 106–107]. Wayward causal chains, freak accidents, and manipulated pathways do not satisfy this condition, even when the agent’s conduct lies among the background circumstances. When both components exhibit the relevant form of responsiveness, the consequence can be said to emanate from the agent in the required sense.

Responsibility also depends on how the outcome is described. When the consequence is specific—this patient’s death, in this way, at this moment—responsibility requires a form of sensitivity: the particular outcome must depend on what the agent did in the right way [9, pp. 99–101]. More general descriptions—that the patient dies—operate differently. Fischer and Ravizza explicitly reject the claim that responsibility for such consequence-universals requires counterfactual dependence: an agent may still be responsible even when the outcome was overdetermined or would have occurred through another sufficient trigger, so long as the actual route from her conduct to the universal remains non-deviant and intelligible [9, pp. 114–121]. Thus, responsibility does not evaporate merely because others also contributed, nor because the outcome might have been unavoidable: what matters is the quality of the agent-anchored route in the actual sequence.

This two-stage structure is well suited to foreseeable-death contexts in medicine, where multiple practitioners, omissions, standing roles, and organisational processes interact. What distinguishes mere causal involvement from attributable contribution is not the agent’s proximity to the outcome nor her capacity to prevent it, but whether her conduct—understood as the expression of her agency—figures within a non-deviant, normatively intelligible sequence leading to the death. This provides the conceptual foundation for the method developed in the next section. To determine, case by case, whether an agent’s behaviour makes a contribution of the relevant kind, a systematic way of testing whether the actual causal sequence exhibits the dependence structure that guidance control identifies is required. The below section develops such a method—counterfactual subtraction—as a practical tool for operationalising consequence-responsibility in clinical and institutional settings.

## Counterfactual subtraction and consequence responsibility

When the outcome is known or reasonably foreseeable—as in many cases of foreseeable death—moral judgement must determine whether the agent’s behaviour figures in the kind of non-deviant, normatively intelligible causal sequence that grounds consequence responsibility. The guidance-control framework specifies what this requires: the behaviour must issue from an owned and moderately reasons-responsive mechanism, and the ensuing outcome must arise from that behaviour along the right kind of route—a causal pathway that is neither deviant nor accidental [9, pp. 119–121]. These conditions capture the features that render a contribution attributable in Fischer and Ravizza’s sense, but they are articulated at a high level of abstraction. In clinical and institutional settings—where multiple agents, omissions, and structural constraints intersect—an operational procedure for determining whether the actual sequence manifests the ‘sensitivity’ relations they describe is needed. Pure counterfactual necessity is too restrictive, excluding responsibility under overdetermination, whereas bare causal involvement is too permissive, sweeping in connections that are wayward or normatively irrelevant. What is required is an intermediate test that can show, in concrete cases, whether the outcome depends in the right way on what the agent did.

This paper therefore adopts counterfactual subtraction as a practical procedure for operationalising the second stage of guidance control. The aim is to determine, in concrete cases, whether an agent’s behaviour forms part of the non-deviant, normatively intelligible causal sequence that grounds consequence responsibility [9, pp. 119–121]. The procedure begins by identifying the elements of the actual sequence that could plausibly contribute to the outcome—acts, omissions, role-indexed decisions, or coordinated institutional moves. These elements are then removed one at a time in a counterfactual scenario while holding fixed the background conditions that constitute the case, such as standing roles, protocols, and structural constraints. The question is whether subtracting a given contribution makes a difference to the outcome in a way that matches Fischer and Ravizza’s account of dependence ‘in the right way’.

Single-contributor cases are the simplest. If the agent’s behaviour is the sole plausible contributor, subtraction proceeds by removing that behaviour and asking whether the outcome would still occur in the same manner. If the outcome remains unchanged, the behaviour is redundant with respect to that particular consequence and does not ground responsibility for that death. If the outcome would not occur—or would occur in a relevantly different way—the behaviour qualifies as a decisive contributor to that particular, since the outcome depends on the behaviour in the way required for consequence responsibility. This mirrors Fischer and Ravizza’s limited sensitivity requirement for consequence-particulars, where responsibility for ‘this death in this manner’ requires dependence along a suitable route [9, pp. 99–101].

Multi-contributor cases require a different application. In many clinical and institutional contexts, two or more contributions jointly guarantee an outcome, so that subtracting any one of them leaves the outcome unchanged. Such cases resemble classical overdetermination. Here, subtraction does not eliminate responsibility: each contributor may still bear consequence responsibility for the universal outcome, provided that in the actual sequence each contribution connects to that universal along a non-deviant, intelligible route [9, pp. 117–121, 230–231]. The test therefore shifts from particular dependence to the broader question emphasised by Fischer and Ravizza: whether the agent’s behaviour forms part of a route that genuinely issues in the universal state of affairs, even where multiple sufficient triggers operate. The absence of counterfactual necessity does not absolve responsibility when the actual sequence retains the right structural connection.

The procedure generalises across acts, omissions, and role-based contributions. An omission can be modelled as a sustained condition guided by an owned, moderately reasons-responsive mechanism; subtraction removes that condition while keeping background constraints fixed [9, p. 119]. Likewise, when the agent’s contribution consists in authorising, enabling, or coordinating within a structured setting, subtraction removes the role-indexed contribution, and responsibility turns on whether the actual organisational route displays the pattern of dependence and non-deviance described by guidance control. In all cases, deviant, accidental, manipulated, or opaque causal chains fail regardless of their counterfactual profile, since such chains do not constitute the ‘right kind of route’ for attributing responsibility [9, pp. 119–121].

Used in this way, counterfactual subtraction does not introduce a new theory of responsibility; it gives practical shape to the second stage of guidance control. By testing how the outcome would shift under the removal of particular contributions, the procedure avoids two familiar distortions. It does not treat individual indispensability as the default threshold for responsibility, since universal-level responsibility may obtain even without counterfactual dependence. And it does not reduce responsibility to mere causal presence, because only routes that are non-deviant and normatively intelligible qualify as attributable [9, pp. 117–121]. Above all, the method makes explicit which changes to the sequence would matter for responsibility, thereby revealing where and how an agent’s contribution figures in the outcome in the way Fischer and Ravizza require. The following sections apply this procedure to cases of killing and letting die in foreseeable-death contexts.

## Distinction between killing and letting die

James Rachels [3] famously argues that there is no intrinsic moral difference between killing and letting die. He begins by targeting the then-standard medical doctrine—endorsed by the American Medical Association—that forbids “mercy killing” while permitting the cessation of extraordinary means; Rachels thinks this pair of permissions/prohibitions is unstable [3, p. 274]. Starting from a terminal-cancer case, he observes that withholding treatment may prolong dying and increase suffering, whereas a lethal injection would be quicker and less painful; once the humanitarian decision ‘not to prolong agony’ is made, active euthanasia can be preferable to passive euthanasia on suffering-minimisation grounds [3, p. 274]. He then presses the Down’s-syndrome/intestinal-obstruction example to show that the doctrine can force life-and-death decisions onto irrelevant grounds: allowing dehydration and infection over days is ‘patently cruel,’ yet an injection that ends life without suffering is ruled out [3, pp. 274–275].

His well-known “Smith and Jones” thought experiment isolates the variable of doing versus allowing. Smith drowns his young cousin to gain an inheritance; Jones, with the same motive and end, stands by as the child drowns “accidentally.” If anything important turned on killing versus letting die as such, Jones’s conduct should be less reprehensible; but, Rachels argues, it is not. Treating ‘I only let him die’ as a defence is, he writes, a ‘grotesque perversion of moral reasoning’ [3, pp. 276–277]. Extending the same lesson to clinical practice, he claims that, when reasons and outcomes are held fixed, a doctor who allows death for humane reasons is in the same moral position as one who administers a lethal injection for humane reasons; if the clinical decision was wrong (for example, the illness was curable), it is equally regrettable regardless of method, and if right, the method ‘is not in itself important’ [3, p. 277]. Rachels further rebuts two replies: the ‘do-nothing’ reply fails because letting die is still something one does—evaluated by the same standards as killing [3, p. 278]—and a focus on ‘cause of death’ does not show a moral difference once death is not judged a greater evil than continued existence in the case at hand [3, p. 278]. His conclusion is emphatic: considered in themselves, there is ‘really no moral difference’ between active and passive euthanasia, even if consequential differences in some cases could make one option preferable to the other [3, p. 279].

Viewed through the lens of moral responsibility, a similar conclusion emerges. The central question is whether an agent who kills or lets die bears equivalent responsibility for the resulting death. This pertains to consequence responsibility, which concerns whether the outcome can be attributed to the agent’s conduct. On the guidance control account, such responsibility does not hinge on abstract distinctions like “killing” or “letting die,” but instead requires that the agent’s conduct (1) issue from a moderately reasons-responsive mechanism that the agent owns, and (2) bring about the consequence via a stable, non-deviant causal pathway—that is, one that is appropriately sensitive to the agent’s action [9, pp. 119–121].

From this perspective, what matters is not whether the conduct is active or passive, but whether it satisfies the conditions for attributing consequence responsibility. If both killing and letting die stem from owned, reasons-responsive mechanisms and each makes a decisive causal contribution to death, the agent may bear equal moral responsibility. If one does and the other does not, the degree of responsibility may differ accordingly.

To illustrate how the guidance control framework applies to the distinction between killing and letting die, I consider three hypothetical cases. In each, the agents’ behaviours are assessed in terms of guidance control and consequence responsibility for the resulting death. In the first case, both killing and letting die make a decisive causal contribution. In the second, neither conduct plays such a role; the death would have occurred regardless. In the third, only the killing satisfies the criteria for consequence responsibility, while the letting die does not. Together, these cases provide a structured basis for evaluating whether and when a moral difference arises between the two forms of conduct.

### Case 1

The first case illustrates how, under the guidance-control framework, killing and letting die can be morally equivalent. Rachels’s “Nasty Cousins” thought experiment provides a familiar template: Smith drowns a child; Jones, with the same motive and intention, refrains from intervening as the child drowns [3, pp. 276–277]. Although one acts and the other omits, their culpability appears indistinguishable.

To align this verdict with guidance control and counterfactual subtraction, the target of assessment must be fixed. At the level of the consequence-universal—that the child dies—counterfactual subtraction brackets competing triggers in the actual case. If we hold Jones’s omission fixed and bracket the accident, death would still obtain (via Jones); if we hold the accident fixed and bracket Jones, death likewise obtains (via the accident). Only when both the omission and the accident are bracketed does the universal change. Each contribution is therefore decisive for the universal, even though neither is individually necessary in the actual world [9].

At the level of the consequence-particular—this accidental drowning in this manner and at this time—the analysis differs: Jones’s omission is redundant with respect to that particular, because the accident alone fixes the manner of death. Guidance control, however, does not require counterfactual dependence on the agent’s conduct for universal-level responsibility, and it rejects the Principle of Possible Prevention as a general test [9]. What matters is that each agent satisfies conduct-responsibility (ownership and moderate reasons-responsiveness) and that the actual route from his conduct to the universal is non-deviant and intelligible. On that basis, Jones’s letting die can ground responsibility for the death as such, even though it is not decisive for the accidental-drowning particular [9].

Seen this way, the structure mirrors the guidance-control treatment of overdetermination: each contributor participates in a minimally sufficient structure for the universal and so bears responsibility for that outcome, while differences at the particular level track how the outcome is realised rather than whether it occurs [9]. Hence—within guidance control—Jones’s letting die is morally equivalent to Smith’s killing with respect to responsibility for the death, even if they differ in their relation to the accidental-drowning particular.

### Case 2

A different kind of moral equivalence between killing and letting die is illustrated by another example discussed by Rachels—one in which neither form of conduct amounts to a decisive contribution to the fatal outcome. In some cases, infants born with Down’s syndrome also suffer from treatable congenital defects, such as intestinal blockage. If the parents decide against corrective surgery, the infant is left to die from dehydration and infection. Rachels argues that, morally, it would make no difference if a physician were instead to administer a lethal injection: since death follows inevitably from the refusal to operate, the mode of the physician’s involvement does not alter the overall moral assessment [3, pp. 274–275].

Viewed through guidance control, this verdict turns on the level at which the outcome is described. At the level of the consequence-universal—that the infant dies—counterfactual subtraction shows that removing the physician’s involvement (whether killing or allowing) does not avert death; only altering the underlying condition or the parents’ choice would do so. The fatal trajectory is fixed by those background determinants, so the physician’s conduct is non-decisive for the universal. By contrast, at the level of a consequence-particular—for example, death by injection at a specific time—the analysis could differ: an active intervention would fix a particular manner of death for which the physician could be responsible. The present claim concerns the universal (“that the infant dies”), where killing and letting die are morally equivalent because neither is decisive for that outcome.

This structure parallels Fischer and Ravizza’s discussion of “Train.” A conductor chooses between two tracks, but the train ends up in the same destination regardless. He may be conduct-responsible for operating the switch, yet he is not consequence-responsible for the train’s arriving at that destination, because the outcome does not counterfactually depend on his choice within the actual sequence [9, pp. 94–95]. Likewise here, the distinction between doing and allowing may appear salient, but under guidance control what matters is whether the agent’s conduct issues from an owned, moderately reasons-responsive mechanism and figures in a non-deviant, intelligible route to the outcome that is sensitive in the appropriate way [9]. For the universal at stake, neither killing nor letting die meets that decisiveness threshold. Accordingly, neither grounds consequence responsibility for the death as such, and the two are morally equivalent in this context.

### Case 3

A sharper moral contrast emerges when one agent meets the conditions for consequence responsibility while another does not. Consider a driver who accidentally strikes an elderly pedestrian, causing injuries that later prove fatal, and a bystander who witnesses the accident but fails to summon help. Although both are present and temporally connected to the outcome, only the driver initiates a non-deviant, intelligible route that culminates in death; provided the conduct issues from an owned, moderately reasons-responsive mechanism, the driver is consequence-responsible not only for the injury but also for the foreseeable fatality that follows within the actual sequence [9, pp. 90, 117–121].

By contrast, the bystander’s letting die does not satisfy the same standard on the level of the consequence for which attribution is sought. Using counterfactual subtraction clarifies the asymmetry. At the level of the consequence-universal—that the victim dies—removing the driver’s action changes the outcome, while removing the bystander’s omission (on the stipulated facts) does not: the victim dies either way. The omission may well be conduct-responsible—it can issue from an owned, moderately reasons-responsive mechanism—but it is non-decisive for the universal because the death does not appropriately depend on it within the actual sequence [9, pp. 117–121] (If timely assistance would have averted death, the subtraction result, and thus the verdict, would differ; the present claim relies on the stipulated inevitability).

This does not deny that omissions can ground consequence responsibility. It marks where the difference lies: in the second-stage requirement that the outcome arise from the agent’s conduct via a suitable route with the right kind of sensitivity, not in the surface contrast between acting and omitting [9, pp. 117–121]. The driver’s behaviour constitutes a morally attributable link to the death as such; the bystander’s failure to intervene, though blameworthy on other grounds (e.g., duties to aid), lacks decisive contribution to that universal outcome in the case as set. Moral difference here tracks responsibility structure—ownership, moderate reasons-responsiveness, and decisive, non-deviant routing—rather than the mere labels “killing” and “letting die” [9].

## Conclusion

This paper has examined the purported distinction between killing and letting die through a responsibility-focused lens, drawing on Fischer and Ravizza’s guidance-control framework to separate responsibility for conduct from responsibility for consequences. On this account, agents are blameworthy for a foreseeable death only when two conditions jointly obtain: their conduct issues from an owned, moderately reasons-responsive mechanism, and the ensuing death arises from that conduct along a non-deviant, normatively intelligible route. Counterfactual subtraction provides a practical way of testing contribution at the second stage, distinguishing between consequence-particulars—sensitive to whether the outcome occurs in this way—and consequence-universals, which may be attributable even in the presence of overdetermination.

Within this framework, the familiar contrast between killing and letting die does not, by itself, settle responsibility judgements. When both forms of conduct meet (or both fail to meet) the relevant responsibility conditions at the level of outcome description, their moral status with respect to the death may converge. When they diverge, the difference traces not the abstract doing/allowing distinction but disparities in ownership, reasons-responsiveness, or decisive causal contribution. These results suggest that much of the perceived moral force of the doing/allowing dichotomy depends on assumptions about responsibility that are rarely made explicit.

The implications of this analysis should be drawn with appropriate caution. The guidance-control framework developed here provides a systematic and practicable structure for retrospective judgement in foreseeable-death contexts, and it aims to clarify how different modes of conduct can bear on responsibility for outcomes. While it does not claim to capture the full “deeper” nature of morality or to provide the only possible normative perspective, it offers a theoretically unified and operationally precise way of analysing responsibility for deaths in end-of-life medicine. How far these insights extend to legal doctrine or clinical governance depends on feasibility-sensitive considerations and on the extent to which the framework’s assumptions align with the practical aims and evidential constraints of those domains [18].

With these qualifications in place, the present account indicates that any moral difference between killing and letting die arises only when responsibility is distributed differently across the two forms of conduct. In cases where responsibility converges, the doing/allowing distinction alone does not furnish an independent basis for different moral or legal judgements. More broadly, a responsibility-centred analysis may help clarify which features of an agent’s involvement matter for retrospective appraisal, while leaving open how such appraisals should interact with broader questions of permissibility, policy, or institutional design.
